# The novel arylindolylmaleimide PDA-66 displays pronounced antiproliferative effects in acute lymphoblastic leukemia cells

**DOI:** 10.1186/1471-2407-14-71

**Published:** 2014-02-06

**Authors:** Christin Kretzschmar, Catrin Roolf, Tina-Susann Langhammer, Anett Sekora, Anahit Pews-Davtyan, Matthias Beller, Moritz J Frech, Christian Eisenlöffel, Arndt Rolfs, Christian Junghanss

**Affiliations:** 1 Department of Hematology/Oncology/Palliative Medicine, Division of Medicine, University of Rostock, Ernst-Heydemann-Str. 6, Rostock 18057, Germany; 2 Leibniz-Institute for Catalysis at the University of Rostock, Albert-Einstein-Str. 29a, Rostock 18059, Germany; 3 Albrecht-Kossel-Institute for Neuroregeneration (Akos), Center for Mental Health, University of Rostock, Gehlsheimerstr. 20, Rostock 18147, Germany; 4 Centogene AG, Schillingallee 68, Rostock 18057, Germany

**Keywords:** Arylindolylmaleimide, Glycogen Synthase Kinase 3β, Acute lymphoblastic leukemia, Apoptosis, Enzyme inhibitors

## Abstract

**Background:**

Prognosis of adult patients suffering from acute lymphoblastic leukemia (ALL) is still unsatisfactory. Targeted therapy via inhibition of deregulated signaling pathways appears to be a promising therapeutic option for the treatment of ALL. Herein, we evaluated the influence of a novel arylindolylmaleimide (PDA-66), a potential GSK3β inhibitor, on several ALL cell lines.

**Methods:**

ALL cell lines (SEM, RS4;11, Jurkat and MOLT4) were exposed to different concentrations of PDA-66. Subsequently, proliferation, metabolic activity, apoptosis and necrosis, cell cycle distribution and protein expression of Wnt and PI3K/Akt signaling pathways were analyzed at different time points.

**Results:**

PDA-66 inhibited the proliferation of ALL cells significantly by reduction of metabolic activity. The 72 h IC50 values ranged between 0.41 to 1.28 μM PDA-66. Additionally, caspase activated induction of apoptosis could be detected in the analyzed cell lines. PDA-66 influenced the cell cycle distribution of ALL cell lines differently. While RS4;11 and MOLT4 cells were found to be arrested in G2 phase, SEM cells showed an increased cell cycle in G0/1 phase.

**Conclusion:**

PDA-66 displays significant antileukemic activity in ALL cells and classifies as candidate for further evaluation as a potential drug in targeted therapy of ALL.

## Background

Acute lymphoblastic leukemia (ALL) is characterized by a poor prognosis in adult patients with a general survival rate of 27 to 54% [1]. In recent years targeted therapeutic approaches such as Imatinib or Rituximab have been developed and implemented successfully in the treatment [2-4]. However, despite of these implementations the prognosis of adult patients remains poor indicating the need for further research in order to identify and evaluate new potential drugs targeting deregulated signaling pathways.

**Figure 1:**
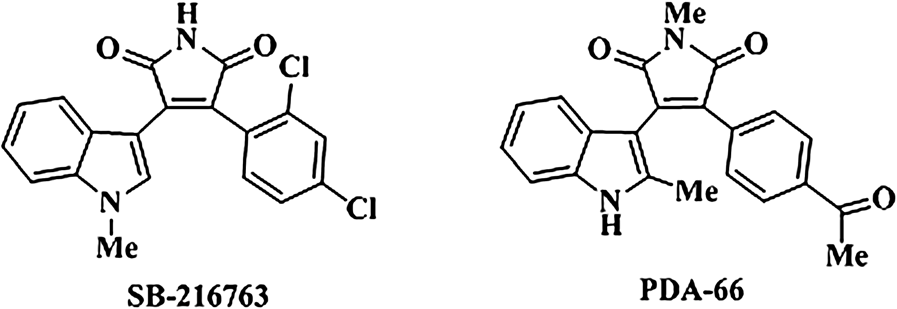
Structural formula of SB-216763 and PDA-66.

Arylindolylmaleimides are a group of synthetic molecules characterized by the conjunction of a maleimide compound with a bicyclic indole ring and a further aromatic structure. PDA-66 is an analogue of the arylindolylmaleimide SB-216763 and was newly synthesized as described by Pews-Davtyan et al. [5]. Both compounds possess similar structural features, but differ in their substitution pattern (Figure 1). In comparison to SB-216763, in PDA-66 the indolyl group is characterized by an unprotected 2-methylindole unit, while the maleimide group is methylated. Notably, the 2,4-dichloro substitution pattern is replaced with 4-acetyl group. Concerning functional activity SB-216763 was shown to inhibit the enzyme activity of Glycogen Synthase Kinase 3β (GSK3β) by 96% at a concentration of 10 μM (IC50: 34.3 nM) in an ATP competitive manner [6] leading to the hypothesis that potential effects of PDA-66 might also be mediated by GSK3β inhibition.

GSK3β is a highly activated serine/threonine kinase in resting cells with normal metabolism [7]. Besides its influence on the glycogen synthesis GSK3β is involved in Wnt/β-catenin and Phosphatidylinositole 3 kinase (PI3K)/Akt signaling antagonizing cell growth and cell cycle progression in both pathways. However, inhibition of GSK3β led to decreased cell growth and increased apoptosis in different tumor cell lines as glioblastoma cells [8], gastrointestinal cancer cells [9,10], ovarian cancer cells [11], medullary thyroid cancer cells [12], pancreatic cancer cells [13] and primary pediatric ALL cells [14]. Joint previous analyses published by Eisenlöffel et al. [15] investigated the influence of PDA-66 in human neuronal progenitor cells (hNPCs) and revealed an inhibitory effect on proliferation and an increased rate of apoptosis. Furthermore, an antiproliferative impact on human lung cancer and glioblastoma cell lines was detected [15].

In this study, we analyzed the biological effects of PDA-66 on B- and T-ALL cell lines and determined the influence on kinase activity of human recombinant GSK3β. Our results show an inhibitory effect on the proliferation and metabolic activity of ALL cells accompanied by an increase in apoptosis and necrosis rates. Furthermore, a minor effect on GSK3β activity could be demonstrated which was not as pronounced as caused by SB-216763.

## Methods

### Inhibitors

PDA-66 was synthesized at the Leibniz Institute for Catalysis (Rostock, Germany) and kindly provided by the Albrecht-Kossel-Institute (Rostock, Germany). SB-216763 was purchased from Sigma (Taufkirchen, Germany). Chemical structures of both substances are displayed in Figure 1. The substances were dissolved in dimethyl sulfoxide (DMSO). The stock solutions (10 mM) were stored at -20°C. For experimental use the drugs were freshly prepared from stock solution.

### Cell lines

The human B-ALL cell lines SEM, RS4;11 and the T-ALL cell lines Jurkat and MOLT4 were purchased from DSMZ (Braunschweig, Germany) and cultured according to manufacturer’s protocol. The corresponding medium was supplemented with 10% heat-inactivated fetal bovine serum (PAA, Pasching, Austria) and 1% penicillin and streptomycin (Biochrom AG, Berlin, Germany). The MOLT4 cells were cultured with medium supplemented with 20% heat-inactivated fetal bovine serum. All cells were maintained at 37°C in 5% CO_2_.

### Treatment of ALL cell lines with PDA-66

Cells (5x10^5^/well) were seeded in 24 well plates (Nunc, Langenselbold, Germany) and incubated for up to 72 h with different concentrations of PDA-66 (0.1 – 10 μM). Treated cells were harvested after 4, 24, 48 and 72 h and used for further analyses. Control cells were cultured in medium containing the same concentration of DMSO as the cells treated with the highest dose of PDA-66.

### Proliferation studies

Cell counts were determined by trypan blue staining. Metabolic activity was analyzed by tetrazolium compound WST-1 (Roche, Mannheim, Germany). In brief, triplicates of cells (5x10^4^/150 μl) were seeded in 96 well plates, treated with different concentrations of PDA-66 and incubated with 15 μl WST-1 for up to 4 h. The mitochondrial dehydrogenases reduce WST-1 to soluble formazan and cause a change of color correlating with the amount of metabolically active cells. Absorbance at 450 nm and a reference wavelength at 620 nm were determined by an ELISA Reader (Anthos, Krefeld, Germany). The absorbance of culture medium with supplemented WST-1 in the absence of cells was used as background control.

### May-Grünwald Giemsa staining

After treatment with 1 μM PDA-66 glass slides were prepared with 3x10^4^ cells with Cytospin 3 centrifuge (Shandon, Frankfurt/Main, Germany). Briefly, slides were incubated 6 min in May-Grünwald solution (Merck, Darmstadt, Germany), washed with tap water, incubated 20 min in Giemsa solution (Merck, Darmstadt, Germany), and washed in tap water again. To evaluate morphological changes of the cells slides were analyzed by Nikon Eclipse E 600 light microscope and imaged with NIS Elements software (Nikon, Düsseldorf, Germany).

### Analyses of apoptosis and necrosis

Apoptosis and necrosis were analyzed by staining the cells with Annexin V FITC (BD Biosciences, Heidelberg, Germany) and Propidium iodide (PI) (Sigma Aldrich, St. Louis, USA). Results were assessed by flow cytometry. Briefly, 5x10^5^ cells were harvested and washed twice (180 g, 10 min, 4°C) with PBS. After resuspending the cells in 100 μl of binding buffer (1×) 4 μl of Annexin V FITC was added and incubated for 15 min at room temperature, respectively. Following addition of 400 μl binding buffer for a final volume of 500 μl the cells were stained with PI (0.6 μg/ml) immediately before measurement. Unstained and single stained cells were included in each experiment as controls. Measurements were performed using FACSCalibur (Becton, Dickinson and Company, Heidelberg, Germany) and data analyzed by CellQuest software (Becton, Dickinson and Company, Heidelberg, Germany).

### Cell cycle analysis

After treatment cells were harvested and washed twice in PBS. Cells were fixed with 70% ethanol and incubated with 1 mg/ml Ribonuclease A (Sigma-Aldrich, St. Louis, USA) for 30 min at 37°C. After washing the cells twice in PBS, they were stained with PI (50 μg/ml) and DNA content was determined by flow cytometry.

### Western blot

Protein extraction and western blot was performed as described previously [16]. Following antibodies were used: rabbit anti-cleaved caspase 3 (5A1E), rabbit anti-caspase 3 (polyclonal), rabbit anti-cleaved PARP (D64E10), rabbit anti-PARP (polyclonal), rabbit anti-cleaved caspase 7 (polyclonal), rabbit anti-caspase 7 (polyclonal), rabbit anti-pAktThr308 (polyclonal), rabbit anti-pAktSer473 (polyclonal), rabbit anti-Akt (polyclonal), rabbit anti-β-catenin (6B3), rabbit anti-pGSK3βSer9 (5B3), rabbit anti-GSK3β (27C10), rabbit anti-p4EBP-1Ser65 (174A9) and rabbit anti-4EBP-1 (polyclonal) (all Cell Signaling, Frankfurt/Main, Germany). Blots were incubated with mouse anti-GAPDH antibody (Invitrogen, Carlsbad, USA) as loading control.

### Kinase activity assay

The kinase activity assay was performed as previously described [17]. Briefly, 20 ng of recombinant human GSK3β (Biomol, Hamburg, Germany) were incubated with the substrate phospho glycogen synthase peptide 2 (pGS2, 25 μM) (Millipore, Billerica, USA), ATP (1 μM) (Cell Signaling, Frankfurt am Main, Germany) and different concentrations of PDA-66 and SB-216763 for 30 min at 30°C. After addition of Kinase-Glo (Promega, Mannheim, Germany) and 10 min of incubation at room temperature the luminescence signal was measured with a Glomax 96 microplate reader (Promega).

### Statistical analysis

Results within each experiment were described using mean ± standard deviation. Significance between control and treated cells was calculated using Student’s t-test. A p-value < 0.05 was considered to be significant. The IC50 values of PDA-66 where determined with SPSS (Version 15) software via probit analysis.

## Results

### PDA-66 inhibits proliferation and metabolic activity of ALL cells

**Figure 2:**
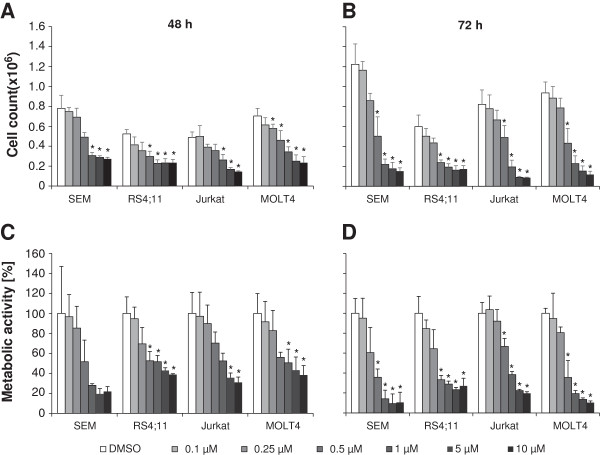
**Treatment with PDA-66 inhibits cell proliferation and metabolic activity.** SEM, RS4;11, Jurkat and MOLT4 cells were incubated with different concentrations of PDA-66. Metabolic activity was determined using WST-1 assay. The results of optical density measurement were expressed as a percentage of the DMSO treated control cells. The two upper diagrams show the results of cell count after PDA-66 treatment after 48 h **(A)** and 72 h **(B)**. The lower diagrams display the influence of PDA-66 on the metabolic activity, respectively **(C, D)**. The proliferation and metabolic activity of all cell lines was suppressed significantly at higher concentrations. Results are displayed as the mean + SD of three independent experiments. *Significant treatment effect vs. DMSO control, α = 0.05.

The influence of PDA-66 on proliferation and metabolic activity in ALL cell lines SEM, RS4;11, Jurkat and MOLT4 was analyzed by incubation with different concentrations of the drug ranging from 0.1 μM to 10 μM for 48 and 72 h, respectively (Figure 2). After 48 h incubation an inhibition of proliferation could be observed (Figure 2A), which was even more distinct after 72 h (Figure 2B). All cell lines showed a significant dose dependent inhibition of proliferation starting at a concentration of 0.5 μM PDA-66.

Likewise proliferation, metabolic activity decreased with increasing concentrations of PDA-66. After 72 h of incubation the metabolic activity was significantly dose dependent reduced in all cell lines starting at a concentration of 0.5 μM PDA-66 (Figure 2D). At this concentration the metabolic activity decreased to 35.7 ± 8.3% in SEM, to 33.3 ± 4.4% in RS4;11, to 66.7 ± 8% in Jurkat and to 35.5 ± 17% in MOLT4 cells compared to control cells treated with DMSO (= 100%). Furthermore, in WST-1 assay the IC50 for PDA-66 in all four cell lines where determined (Table 1). The IC50 values ranged from 0.41 μM in SEM cells to 1.28 μM in Jurkat cells after 72 h of incubation.

**Table 1:** IC50 values of PDA-66 in WST-1 assay

	**IC50 [μM]**	
	**48 h**	**72 h**
**SEM**	0.85	0.41
**RS4;11**	2.14	0.74
**Jurkat**	2.32	1.28
**MOLT4**	2.41	0.52

The incubation of ALL cell lines with higher dosages of PDA-66 (0.5 μM or more) led to a decrease in cell numbers below the amount of seeded cells (5x10^5^). This result indicates besides an inhibition of cell proliferation also an induction of cell death.

### PDA-66 influences morphology as well as cell cycle progression and induces apoptosis

**Figure 3:**
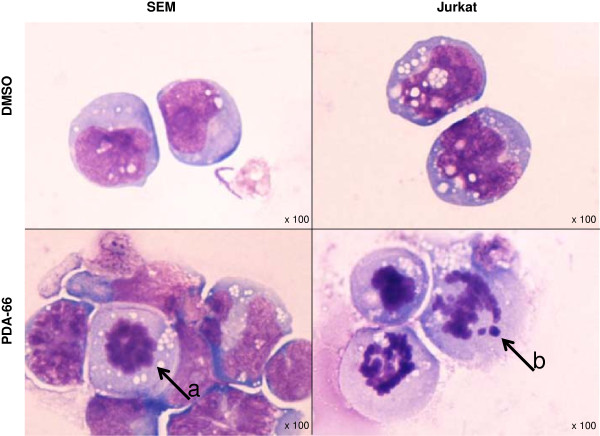
**Light microscopy reveals karyorrhectic morphology after PDA-66 treatment in SEM and Jurkat cells.** Cytospins of SEM and Jurkat cells were stained with Pappenheim method after 48 h incubation with 1 μM PDA-66 and DMSO, respectively. Representative pictures are displayed. The upper pictures show SEM and Jurkat cells after DMSO treatment, the lower ones show PDA-66 treated cells. After treatment with PDA-66 an increased amount of cells with chromatin condensation **(black arrow a)** and karyorrhexis **(black arrow b)** could be observed along with more cell debris.

To evaluate possible morphological changes cells were treated with 1 μM of PDA-66 for 48 h and analyzed by light microscopy. All four cell lines showed similar changes in morphology after PDA-66 treatment compared to DMSO treated control cells. Exemplarily, effects in SEM and Jurkat cells are shown in Figure 3. In contrast to DMSO treated control cells the incubation of 1 μM PDA-66 led to condensation of chromatin in the nucleus, karyorrhexis and an increasing amount of vacuoles and cell debris after 48 h of treatment. Condensated chromatin points to an induction of apoptosis or cell cycle arrest in the analyzed cell lines.

**Figure 4:**
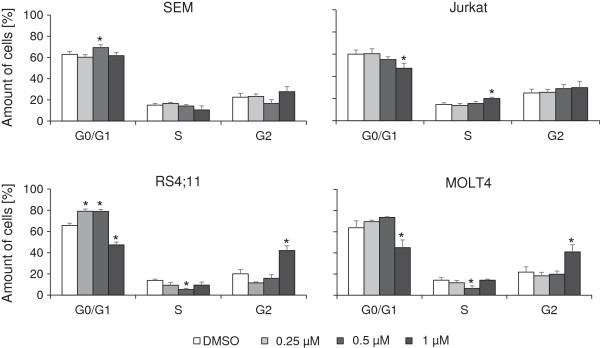
**PDA-66 leads to cell cycle arrest in G2 phase in RS4;11 and MOLT4 cells.** ALL cell lines were incubated for 48 h with PDA-66 and cell cycle distribution was determined using Propidium iodide staining. On the left side the amount of cells in the different phases of cell cycle is shown for the two B-ALL cell lines SEM and RS4;11. On the right side the results for the T-ALL cell lines Jurkat and MOLT4 are displayed, respectively. G2 arrest could be detected in RS4;11 and MOLT4 cells. Treatment of Jurkat cells induced a decrease of cells in G0/G1 phase in favor of cells in S phase. Results are displayed as the mean + SD of three independent experiments. *Significant treatment effect vs. DMSO control, α = 0.05.

Cell cycle analysis was performed by PI staining and flow cytometrical measurement. The treatment with PDA-66 for 48 h influenced the four cell lines in different manner (Figure 4). SEM cells showed a significant increase in the amount of cells in G0/G1 after incubation with 0.5 μM (DMSO control: 62.8 ± 2.8%; 0.5 μM PDA-66: 69.3 ± 2.7%) whereas 1 μM did not affect the cell cycle significantly. RS4;11 and MOLT4 cells were characterized by a significant G2 arrest after treatment with 1 μM PDA-66. The amount of RS4;11 and MOLT4 cells in G2 phase increased from 20.1 ± 3.9% and 21.9 ± 4.9% after incubation with DMSO to 42.1 ± 4.4% and 41.0 ± 5.8% after 1 μM PDA-66 treatment. This was associated with a significant decrease in G0/G1 phase (RS4;11 and MOLT4: 65.7 ± 2.1% and 63.7 ± 6.6% in control; 47.3 ± 2.7% and 45.0 ± 7.3% after treatment with 1 μM PDA-66). On the other hand lower concentrations led to significant increase of cells in G0/G1 phase. Jurkat cells showed a significant decrease in G0/G1 phase (from 60.0 ± 3.7% in control to 47.3 ± 4.3% with PDA-66) and an increase in S phase (from 14.6 ± 1.5% in control to 20.0 ± 1.1% with PDA-66) after incubation with 1 μM PDA-66. The analyses of cell cycle after longer incubation intervals interfered with high rates of apoptosis and necrosis (data not shown).

**Figure 5:**
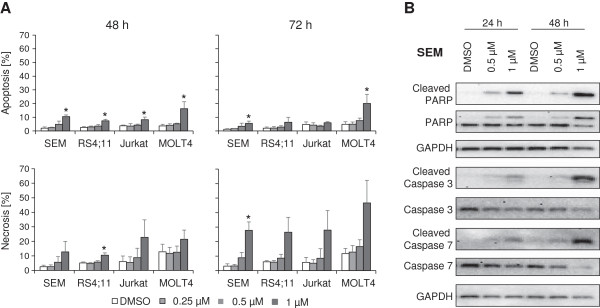
**Treatment with PDA-66 induces apoptosis via cleavage of caspases. (A)** Cells were treated with PDA-66 for up to 72 h and stained with Annexin V FITC and Propidium iodide (PI). Rates of early apoptotic (FITC^+^, PI^-^) and late apoptotic and necrotic (FITC^+^, PI^+^) cells were measured by flow cytometry. The upper diagrams display the rate of apoptotic cells after 48 h (left) and 72 h (right). The lower diagrams show the results for necrosis measurement, respectively. Significant induction of apoptosis could be observed in all cell lines after 48 h of incubation as well as tendential induction of necrosis at both points of time. Results are displayed as the mean + SD of three independent experiments. *Significant treatment effect vs. DMSO control, α = 0.05. **(B)** Cells were treated with different concentrations of PDA-66 and total cell lysates (25 μg) were analyzed by Western blot to detect cleavage of Caspase 3, 7 and PARP. GAPDH was used as loading control. Exemplary results of PDA-66 treated SEM cells after 24 and 48 h are displayed. Induction of apoptosis was confirmed by an increase of the cleaved forms of Caspase 3, 7 and PARP.

The effect of PDA-66 on apoptosis and necrosis rates was determined by flow cytometric analysis after 48 and 72 h of incubation and further analysed by western blot after 24 and 48 h, respectively (Figure 5). After 48 h of incubation all PDA-66 treated cell lines showed a significant increase in apoptosis compared to control cells (SEM: 2.1 ± 0.9% to 10.5 ± 1.3%; RS4;11: 2.5 ± 0.7% to 7.4 ± 1.1%; Jurkat: 3.8 ± 0.6% to 8.3 ± 1.9%; MOLT4: 3.7 ± 1.2% to 16.3 ± 5.1%). After 72 h a similar tendency could be observed, but only deviations in SEM and MOLT4 cells where significant (SEM: 1.3 ± 0.4% to 5.6 ± 1.6%; RS4;11: 2.1 ± 0.9% to 6.4 ± 3.6%; Jurkat: 4.7 ± 1.9% to 6.1 ± 0.7%; MOLT4: 4.9 ± 1.9% to 20.1 ± 6.6%).

All cells showed a non significant increase in necrosis after 48 and 72 h incubation with 1 μM PDA-66. After 72 h incubation necrosis rate rose in SEM cells from 3.1 ± 1.6% to 27.8 ± 5.81%, in RS4;11 cells from 6.1 ± 0.8% to 26.5 ± 10.2%, in Jurkat cells from 5.7 ± 3.5% to 28.0 ± 13.4% and in MOLT4 cells from 11.7 ± 3.6% to 46.7 ± 15.6% (Figure 5A). Analysis via western blot showed an apoptosis induction in all cell lines. Treatment with PDA-66 induced cleavage of caspases 3 and 7 and PARP 48 h after addition of PDA-66. In Figure 5B results of SEM cells are displayed exemplarily.

### PDA-66 influences protein expression of 4EBP-1, but not β-catenin

**Figure 6:**
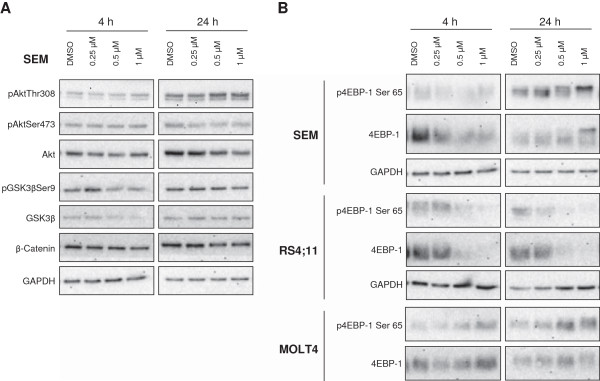
**PDA-66 does not influence expression of proteins of Wnt/β-catenin pathway but alters expression of 4EBP-1.** After treatment with PDA-66 and DMSO, respectively, cells were lyzed and protein expression analyzed with Western blot. **(A)** Exemplary results of PDA-66 treated SEM cells after 4 and 24 h are displayed. No influence on expression of total GSK3β and the total form of Akt could be noticed. PhosphoGSK3βSer9 seemed decreased at higher PDA-66 concentrations after 4 h. No influence on the amount of β-catenin was observed. **(B)** Exemplary results of SEM, RS4;11 and MOLT4 cells are displayed. In SEM and RS4;11 cells a decrease of 4EBP-1 and p4EBP-1Ser65 was detectable, in contrast MOLT4 cells showed an increased expression of p4EBP-1Ser65 after PDA-66 treatment.

In order to characterize the effects of PDA-66 on PI3K/Akt and Wnt/β-catenin pathways we performed western blot analysis. The time points of western blot analysis were shifted to 4 and 24 h as effects on protein level are expected to be detectable earlier compared to the effects on the whole cell. The incubation with PDA-66 showed no detectable influence on the expression of β-catenin, total GSK3β and total Akt at both time points (Figure 6A). However, an increase of pAktThr308 could be detected in SEM cells after an incubation of 24 h, though not accompanied by an increase of pAktSer473. Furthermore, in SEM cells a decrease of pGSK3βSer9 was observed after 4 h. However, no influence on the total form of β-catenin was detectable. Nevertheless, there was an influence of PDA-66 on the expression of 4EBP-1 and p4EBP-1Ser65. SEM, RS4;11 and Jurkat cells showed a decrease of the phosphorylated as well as the total form of 4EBP-1 after an incubation of 4 and 24 h. In contrast, MOLT4 cells displayed an increase of the phosphorylated form at these points of time (Figure 6B).

### PDA-66 does not inhibit kinase activity of recombinant GSK3β as distinct as SB-216763

**Figure 7:**
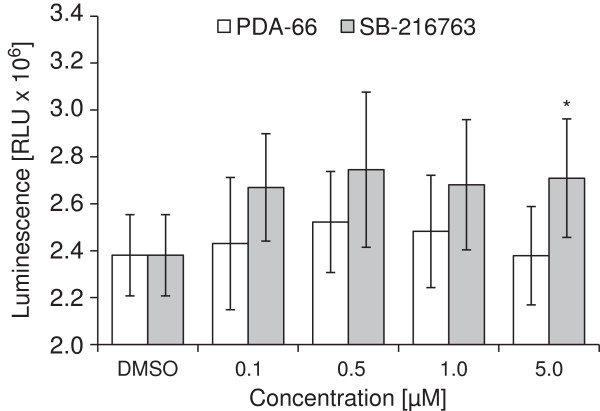
**PDA-66 does not inhibit GSK3β kinase activity.** Recombinant human GSK3β was incubated with pGS2, ATP and different concentrations of PDA-66 and SB-216763. While treatment with the known GSK3β inhibitor SB-216763 lead to a stable reduction of enzyme activity with significant alterations at 5 μM PDA-66 showed only a slight inhibitory potential. Results are displayed as the mean ± SD of five independent experiments. In each experiment the concentrations of PDA-66 and the control were tested with 4 replicates. RLU = relative luminescence units. *Significant treatment effect vs. DMSO control, α = 0.05.

The effect of PDA-66 on the GSK3β enzyme activity was determined by incubation with the specific substrate pGS2, PDA-66 or SB-216763 and ATP. The following addition of Kinase-Glo reagent converts the remaining ATP into a luminescence signal which correlates with enzyme inhibition. SB-216763 demonstrated a stable inhibition of GSK3β at concentrations from 0.1 to 5 μM which was statistically significant at 5 μM (Figure 7). Compared to this PDA-66 showed a less pronounced inhibition of enzyme activity at concentration from 0.1 to 1 μM which were not significant.

## Discussion

The prognosis of ALL in adult patients is still poor and requires further research for new therapeutic approaches. In this study we could demonstrate for the first time a pronounced antiproliferative effect of the novel arylindolylmaleimide PDA-66 on different B and T ALL cell lines. We investigated the influence of PDA-66 on ALL cells in respect of proliferation, metabolic activity, morphology, apoptosis, cell cycle arrest, and activation of PI3K/Akt and Wnt/β-catenin signaling pathways. Furthermore, the effect on kinase activity of GSK3β was determined.

PDA-66 was recently synthesized and described as an analogue to SB-216763, which is a known GSK3β inhibitor [6]. The inhibition of this kinase has been extensively examined in various neoplastic cells types and demonstrated an attenuated proliferation in malignant cells [9-14]. Investigating the influence of PDA-66 on the enzyme activity of human recombinant GSK3β we found a minor inhibition *in vitro* which was much less distinct and not significant compared to our results obtained with SB-216763. While the basic molecular structure of SB-216763 and PDA-66 is the same, both compounds differ in their substitution patterns. In comparison to SB-216763, PDA-66 is characterized by an unprotected 2-methylindole group and a methylated maleimide group. Additionally, the 2,4-dichloro substitution pattern is replaced with a 4-acetyl group in PDA-66. These structural changes are supposed to be key in the reduced capacity of PDA-66 to inhibit GSK3β.

The influence of PDA-66 on GSK3β activity including other key enzymes of Wnt/β-catenin and PI3K/Akt signaling pathways was also investigated by western blot. Affirming the results obtained by kinase activity assay, we found no enhanced activation of the Wnt/β-catenin pathway. Considering the role of GSK3β in Wnt signaling an increase of β-catenin would have been expected when inhibiting GSK3β. Furthermore, no effect on the protein expression of GSK3β and no distinct activation of Akt were detectable. In the PI3K/Akt signaling pathway GSK3β acts downstream of Akt [18], although Takada et al. demonstrated that the TNF induced activity of Akt is dependent on GSK3β [19], indicating a possible feedback loop. In the herein analyzed SEM cells a slight increase in pAktThr308 could be observed. However, there was no detectable increase in pAktSer473, which is primarily responsible for activation of Akt [20].

Interestingly, PDA-66 influenced the phosphorylation status and the total amount of protein of 4EBP-1 at 4 and 24 h after treatment. 4EBP-1 is a downstream target of mTOR, which is inhibited by GSK3β via phosphorylation of TSC2 [21]. The phosphorylation and concomitant inactivation of 4EBP-1 by mTOR leads to disaggregation of 4EBP-1 from eIF4F, a translation initiation factor [22]. Walsh and Mohr demonstrated that the phosphorylation of 4EBP-1 leads to its proteasomal degradation [23]. In our study the effect of PDA-66 on the amount of 4EBP-1 was ambiguous. SEM, RS4;11 and Jurkat cells displayed a reduced expression whereas MOLT4 cells showed an enhanced amount of 4EBP-1 protein. A decreased level of protein can be caused by enhanced degradation or reduced transcription and translation, respectively. The expression of 4EBP-1 was shown to be positively regulated by transcription factor ATF-4, which is activated by JNK signaling in murine pancreatic beta-cells [24]. Furthermore, JNK is a mitogen activated protein kinase and therefore member of a complex cascade [25]. An effect of PDA-66 on one of these proteins might also influence the activation of ATF-4 and hence 4EBP-1 expression. Nevertheless, there is a probable influence of PDA-66 on other enzymes and cascades.

Although there was no influence on GSK3β detectable, we hypothesized that the application of PDA-66 could nevertheless induce comparable antiproliferative effects in ALL cancer cells as SB-216763 due to the similar basic molecular structure. Notably, PDA-66 treated ALL cells showed a significant decrease in cell count and metabolic activity which was more distinct than results obtained in standard reference experiments with SB-216763 (data not shown). Furthermore the treatment with PDA-66 led to morphological changes like condensation of chromatin and karyorrhexis which can be attributed to the detected induction of apoptosis as well as cell cycle alterations.

Our studies indicate different influences on cell cycle in the analyzed ALL cell lines after 48 h incubation with PDA-66. Concentrations of 0.25 and 0.5 μM PDA-66 led to an increase of cells in the G0/G1 phase whereas treatment with 1 μM was followed by decrease of G0/G1 phase and a significant increase in G2 phase in RS4;11 and MOLT4 cells. Jurkat cells also showed a decreasing amount of cells in G0/G1 phase, whereas an increase was detected in SEM cells after incubation with 0.5 μM PDA-66. In a previous joint study presented by Eisenlöffel et al. it could be displayed that PDA-66 treatment at comparable concentrations as used in these analyses leads to mitotic arrest in the G2/M phase in hNPCs [15]. This effect on cell cycle was caused by inhibition of microtubule polymerization [15]. Treatment of hNPCs with PDA-66 also led to an attenuated proliferation and an increased rate of apoptosis [15]. An antiproliferative effect was also demonstrated in human cell lines of lung cancer and glioblastoma [15]. Similar results were obtained in our study. The analyzed ALL cells showed a significant increase of apoptosis 48 h after treatment with PDA-66.

## Conclusion

We demonstrated for the first time a significant and pronounced antiproliferative influence of PDA-66 on ALL cells. In addition, we showed an induction of apoptosis via cleavage of caspases as well as suppression of metabolic activity. While there was an effect on cell cycle progression, no influence on the Wnt/β-catenin signaling pathway was observed. The investigation of enzyme activity of GSK3β showed a minor inhibitory effect compared to the analogue substance SB-216763. Nevertheless, the herein observed anti-tumoral potential in ALL and the previous seen effects in neoplastic tissues classify PDA-66 as a promising novel therapeutic agent candidate. Consequently, the detailed analyses of PDA-66 mediated effects should be further elucidated and validated *in vivo* as a base for a perspective therapeutic consideration.

## Abbreviations

4EBP-1: Eukaryotic initiation factor 4E binding protein-1; ALL: Acute lymphoblastic leukemia; ATF-4: Activating transcription factor 4; DMSO: Dimethyl sulfoxide; GSK3β: Glycogen synthase kinase 3β; hNPCs: Human neuronal progenitor cells; IC50: Half maximal inhibitory concentration; JNK: C-Jun N-terminal kinase; PARP: Poly (ADP)-ribose polymerase; pGS2: Phospho glycogen synthase peptide 2; PI3K: Phosphatidylinositole 3 kinase; TNF: Tumor necrosis factor.

## Competing interests

The authors declare that they have no competing interests.

## Authors’ contributions

CK performed all experiments, participated in study design, partial data analysis and interpretation, partial manuscript drafting. CR performed all experiments, participated in study design, partial data analysis and interpretation, partial manuscript drafting. TSL helped carrying out western blot experiments. AS helped carrying out cell cultivation, proliferation studies and analyses of apoptosis, necrosis and cell cycle. APD developed new substance PDA-66 and partial manuscript editing. MB developed new substance PDA-66 and partial manuscript editing. MJF participated in drug development, partial manuscript editing. CE participated in drug development partial manuscript editing. AR participated in drug development and partial manuscript editing. CJ principal study design, participated in the design of the paper and finalization. All authors read and approved the final manuscript.

## Pre-publication history

The pre-publication history for this paper can be accessed here:

http://www.biomedcentral.com/1471-2407/14/71/prepub

## References

[B1] Gokbuget N Hoelzer D Treatment of adult acute lymphoblastic leukemia Semin Hematol 2009 46 64 75 19100369 10.1053/j.seminhematol.2008.09.003

[B2] Thomas DA Faderl S O’Brien S Bueso-Ramos C Cortes J Garcia-Manero G Giles FJ Verstovsek S Wierda WG Pierce SA Shan J Brandt M Hagemeister FB Keating MJ Cabanillas F Kantarjian H Chemoimmunotherapy with hyper-CVAD plus rituximab for the treatment of adult Burkitt and Burkitt-type lymphoma or acute lymphoblastic leukemia Cancer 2006 106 1569 1580 16502413 10.1002/cncr.21776

[B3] Ottmann OG Wassmann B Pfeifer H Giagounidis A Stelljes M Duhrsen U Schmalzing M Wunderle L Binckebanck A Hoelzer D Imatinib compared with chemotherapy as front-line treatment of elderly patients with Philadelphia chromosome-positive acute lymphoblastic leukemia (Ph + ALL) Cancer 2007 109 2068 2076 17429836 10.1002/cncr.22631

[B4] Vignetti M Fazi P Cimino G Martinelli G Di Raimondo F Ferrara F Meloni G Ambrosetti A Quarta G Pagano L Rege-Cambrin G Elia L Bertieri R Annino L Foa R Baccarani M Mandelli F Imatinib plus steroids induces complete remissions and prolonged survival in elderly Philadelphia chromosome-positive patients with acute lymphoblastic leukemia without additional chemotherapy: results of the Gruppo Italiano Malattie Ematologiche dell’Ad Blood 2007 109 3676 3678 17213285 10.1182/blood-2006-10-052746

[B5] Pews-Davtyan A Tillack A Ortinau S Rolfs A Beller M Efficient palladium-catalyzed synthesis of 3-aryl-4-indolylmaleimides Org Biomol Chem 2008 6 992 997 18327323 10.1039/b719160j

[B6] Coghlan MP Culbert AA Cross DA Corcoran SL Yates JW Pearce NJ Rausch OL Murphy GJ Carter PS Roxbee Cox L Mills D Brown MJ Haigh D Ward RW Smith DG Murra KJ Reith AD Holder JC Selective small molecule inhibitors of glycogen synthase kinase-3 modulate glycogen metabolism and gene transcription Chem Biol 2000 7 793 803 11033082 10.1016/s1074-5521(00)00025-9

[B7] Kockeritz L Doble B Patel S Woodgett JR Glycogen synthase kinase-3–an overview of an over-achieving protein kinase Current drug targets 2006 7 1377 1388 17100578 10.2174/1389450110607011377

[B8] Korur S Huber RM Sivasankaran B Petrich M Morin PJ Hemmings BA Merlo A Lino MM GSK3beta regulates differentiation and growth arrest in glioblastoma PloS One 2009 4 e7443 19823589 10.1371/journal.pone.0007443 PMC2757722

[B9] Mai W Kawakami K Shakoori A Kyo S Miyashita K Yokoi K Jin M Shimasaki T Motoo Y Minamoto T Deregulated GSK3beta sustains gastrointestinal cancer cells survival by modulating human telomerase reverse transcriptase and telomerase Clin Canc Res 2009 15 6810 6819 10.1158/1078-0432.CCR-09-0973 19903789

[B10] Ghosh JC Altieri DC Activation of p53-dependent apoptosis by acute ablation of glycogen synthase kinase-3beta in colorectal cancer cells Clin Canc Res 2005 11 4580 4588 10.1158/1078-0432.CCR-04-2624 15958644

[B11] Cao Q Lu X Feng Y Glycogen synthase kinase-3beta positively regulates the proliferation of human ovarian cancer cells Cell Res 2006 16 671 677 16788573 10.1038/sj.cr.7310078

[B12] Kunnimalaiyaan M Vaccaro AM Ndiaye MA Chen H Inactivation of glycogen synthase kinase-3beta, a downstream target of the raf-1 pathway, is associated with growth suppression in medullary thyroid cancer cells Mol Cancer Ther 2007 6 1151 1158 17363508 10.1158/1535-7163.MCT-06-0665

[B13] Ougolkov AV Fernandez-Zapico ME Savoy DN Urrutia RA Billadeau DD Glycogen synthase kinase-3beta participates in nuclear factor kappaB-mediated gene transcription and cell survival in pancreatic cancer cells Cancer research 2005 65 2076 2081 15781615 10.1158/0008-5472.CAN-04-3642

[B14] Hu Y Gu X Li R Luo Q Xu Y Glycogen synthase kinase-3beta inhibition induces nuclear factor-kappaB-mediated apoptosis in pediatric acute lymphocyte leukemia cells J Exp Clin Cancer Res 2010 29 154 21110852 10.1186/1756-9966-29-154 PMC3002327

[B15] Eisenlöffel C Schmöle AC Pews-Davtyan A Brennführer A Kuznetsov SA Hübner R Frech S Schult C Junghanss C Beller M Rolfs A Frech MJ Interference of a novel indolylmaleimide with microtubules induces mitotic arrest and apoptosis in human progenitor and cancer cells Biochem Pharmacol 2013 85 6 763 771 23274302 10.1016/j.bcp.2012.12.013

[B16] Schult C Dahlhaus M Ruck S Sawitzky M Amoroso F Lange S Etro D Glass A Fuellen G Boldt S Wolkenhauer O Neri LM Freund M Junghanss C The multikinase inhibitor Sorafenib displays significant antiproliferative effects and induces apoptosis via caspase 3, 7 and PARP in B- and T-lymphoblastic cells BMC Cancer 2010 10 560 20950443 10.1186/1471-2407-10-560 PMC2972283

[B17] Schmole A Brennfuhrer A Karapetyan G Jaster R Pews-Davtyan A Hubner R Ortinau S Beller M Rolfs A Frech MJ Novel indolylmaleimide acts as GSK-3beta inhibitor in human neural progenitor cells Bioorg Med Chem 2010 18 6785 6795 20708937 10.1016/j.bmc.2010.07.045

[B18] Vivanco I Sawyers CL The phosphatidylinositol 3-Kinase AKT pathway in human cancer Nat Rev Cancer 2002 2 489 501 12094235 10.1038/nrc839

[B19] Takada Y Fang X Jamaluddin MS Boyd DD Aggarwal BB Genetic deletion of glycogen synthase kinase-3beta abrogates activation of IkappaBalpha kinase, JNK, Akt, and p44/p42 MAPK but potentiates apoptosis induced by tumor necrosis factor J Biol Chem 2004 279 39541 39554 15252041 10.1074/jbc.M403449200

[B20] Park S Chapuis N Tamburini J Bardet V Cornillet-Lefebvre P Willems L Green A Mayeux P Lacombe C Bouscary D Role of the PI3K/AKT and mTOR signaling pathways in acute myeloid leukemia Haematologica 2010 95 819 828 19951971 10.3324/haematol.2009.013797 PMC2864389

[B21] Inoki K Ouyang H Zhu T Lindvall C Wang Y Zhang X Yang Q Bennett C Harada Y Stankunas K Wang C He X MacDougald OA You M Williams BO Guan K TSC2 integrates Wnt and energy signals via a coordinated phosphorylation by AMPK and GSK3 to regulate cell growth Cell 2006 126 955 968 16959574 10.1016/j.cell.2006.06.055

[B22] Morley SJ Coldwell MJ Clemens MJ Initiation factor modifications in the preapoptotic phase Cell Death Differ 2005 12 571 584 15900314 10.1038/sj.cdd.4401591

[B23] Walsh D Mohr I Phosphorylation of eIF4E by Mnk-1 enhances HSV-1 translation and replication in quiescent cells Gene Dev 2004 18 660 672 15075293 10.1101/gad.1185304 PMC387241

[B24] Tominaga R Yamaguchi S Satake C Usui M Tanji Y Kondo K Katagiri H Koa Y Ishihara H The JNK pathway modulates expression and phosphorylation of 4E-BP1 in MIN6 pancreatic beta-cells under oxidative stress conditions Cell Biochem Funct 2010 28 387 393 20589738 10.1002/cbf.1667

[B25] Saadeddin A Babaei-Jadidi R Spencer-Dene B Nateri AS The links between transcription, beta-catenin/JNK signaling, and carcinogenesis Mol Cancer Res 2009 7 1189 1196 19671687 10.1158/1541-7786.MCR-09-0027

